# 

**Published:** 1887-02

**Authors:** 


ANNUAL COMMENCEMENT OF THE UNIVERSITY OF MARYLAND, DENTAL DEPARTMENT.—The commencement exercises for 1887 will be held at the Academy of Music, Baltimore, on the 16th day of March next, at 12 M.

The contest of the Graduating Class for the Annual Prizes, will take place in the Dental Infirmary on March 15th, commencing at 10 A. M.

This commencement will be held separately from that of the Medical Department. It has been the custom heretofore, to hold the commencement exercises of the two departments at the same time and place, but the large classes in both necessitates a change in this respect, and hereafter separate commencements will be held.

### *To Readers and Correspondents!*

Communications intended for publication, and exchange journals and papers should be addressed to the EDITOR OF THE AMERICAN JOURNAL OF DENTAL SCIENCE, 259 N. Eutaw St., Baltimore, Md. All letters relating to the business of the JOURNAL, or containing cash remittances, to be directed to Messrs. SNOWDEN & COWMAN. Articles for publication should be received by the editor at least three weeks previous to the first month on which the number in which it is designed for them to appear, is issued.

☞ The AMERICAN JOURNAL OF DENTAL SCIENCE will contain fifty pages of reading matter in each number, making a volume of about

*Six Hundred* pages,

EXCLUSIVE OF ADVERTISEMENTS.

